# TbUNC119 and Its Binding Protein Complex Are Essential for Propagation, Motility, and Morphogenesis of *Trypanosoma brucei* Procyclic Form Cells

**DOI:** 10.1371/journal.pone.0015577

**Published:** 2010-12-22

**Authors:** Shigeru Ohshima, Mitsuko Ohashi-Suzuki, Yutaka Miura, Yoshisada Yabu, Noriko Okada, Nobuo Ohta, Takashi Suzuki

**Affiliations:** 1 Department of Core Laboratory, Nagoya City University Graduate School of Medical Sciences, Nagoya, Japan; 2 Department of Molecular Parasitology, Nagoya City University Graduate School of Medical Sciences, Nagoya, Japan; 3 Department of Immunology, Nagoya City University Graduate School of Medical Sciences, Nagoya, Japan; 4 Division of Public Health, Department of International Health Development, Graduate School of Tokyo Medical and Dental University, Tokyo, Japan; 5 Department of Molecular Neurology, Nagoya City University Graduate School of Medical Sciences, Nagoya, Japan; Federal University of São Paulo, Brazil

## Abstract

Flagellum-mediated motility of *Trypanosoma brucei* is considered to be essential for the parasite to complete stage development in the tsetse fly vector, while the mechanism by which flagellum-mediated motility is controlled are not fully understood. We thus compared *T. brucei* whole gene products (amino acid sequence) with *Caenorhabditis elegans* UNC (uncoordinated) proteins, in order to find uncharacterized motility-related *T. brucei* genes. Through *in silico* analysis, we found 88 gene products which were highly similar to *C. elegans* UNC proteins and categorized them as TbCEUN (*T. brucei* gene products which have high similarity to *C. elegans* UNC proteins). Approximately two thirds of the 88 TbCEUN gene products were kinesin-related molecules. A gene product highly similar to *C. elegans* UNC119 protein was designated as TbUNC119. RNAi-mediated depletion of TbUNC119 showed no apparent phenotype. However, knock-down analysis of both TbUNC119 and its binding protein (TbUNC119BP) which was found by yeast two-hybrid analysis showed characteristic phenotypes, including reduced motility, morphological change (extended cell shape), and cellular apoptosis. Based on the observed phenotypes, possible function of the TbUNC119 and TbUNC119BP is discussed.

## Introduction

African trypanosomes (e.g., *Trypanosoma brucei* and related subspecies) are uniflagellated protozoan parasites that cause African trypanosomiasis in humans and nagana disease in wild and domestic animals [1]. *T. brucei* is transmitted to the bloodstream of mammalian hosts through the bite of an infected tsetse fly. The organisms multiply in the host's bloodstream and eventually spread to the central nervous system, where they initiate a cascade of events that results in fatal sleeping sickness [1]. Thus, the pathogenic feature of the disease is directly related to parasite motility. Indeed coordinated motility is crucial for disease pathogenesis in mammalian hosts [2].

The highly complicated stage development of *T. brucei* has been characterized in the tsetse fly vector [3]. After a tsetse fly ingests *T. brucei* from the bloodstream of an infected host, the trypanosomes differentiate into dividing procyclic forms and establish an infection in the tsetse fly midgut [4]. The parasites then differentiate into dividing elongated epimastigotes [3], [4], which migrate to the salivary gland where they stop dividing, become shorter, attach to the gland epithelium, and differentiate into metacyclic forms [3], [4]. The metacyclic forms detach from the epithelium, move to the proboscis, and prepare to propagate in the mammalian bloodstream [3], [4]. Parasite motility is considered to be essential for completion of this stage development process within the tsetse fly vector.

Since *T. brucei* parasites migrate by flagellum organelle, there is considerable interest in clarifying its mechanisms of flagellum-mediated motility [3], [5]. In addition to functional analysis of individual flagellum genes, the proteomic study by Baron *et al.* for *T. brucei* whole flagellum proteins is under way [6]. Knock-down analysis of flagellum-protein genes uncovered the important roles of these proteins for propagation of *T. brucei* cells [6]. However, the mechanism of *T. brucei* flagellum-mediated motility is still not fully understood. In particular, little is known about the relationship between motility mechanisms and stage development. It is therefore important to identify more proteins involved in *T. brucei* motility.

The free-living nematode, *Caenorhabditis elegans*, is a model organism that has been used to study development, signal transduction, and motility [7]. Genes required for correct motility of *C. elegans* has been referred to as “UNC” genes, since mutations of these genes caused *unc*oordinated locomotion [7]. Currently, there are 112 *C. elegans* UNC genes in WormBase Release WS206 (http://www.wormbase.org/).

Although mechanisms by which motility is generated in *T. brucei* and *C. elegans* are different, motility is crucial to survival of both species. We thus compared *T. brucei* whole gene products (amino acid sequence) with *C. elegans* UNC proteins *in silico* in order to find uncharacterized motility-related *T. brucei* genes. Using this method, we found 88 genes that were highly similar to genes encoding *C. elegans* UNC proteins, and categorized them as TbCEUN (*T. brucei* gene products which have high similarity to *C. elegans* UNC proteins). Among the 88 TbCEUN, we found a gene product with a high degree of similarity to *C. elegans* UNC119, and designated it as TbUNC119.

The *C. elegans* UNC119 (CeUNC119) gene was cloned as a gene expressed in the nervous system. CeUNC119 is required for coordinated locomotion of *C. elegans* and proper development of the worm nervous system, including axonal branching and fasciculation [8], [9], [10]. The human homologue gene product, HsUNC119, is an adaptor-signaling molecule, which regulates activation of tyrosine kinases in T cells, eosinophils, and fibroblasts [11], [12], [13]. HsUNC119 also plays an important role in the photoreceptor synapses of the retina [14]. In the present study, we focused on TbUNC119 and analyzed its function in *T. brucei* in terms of parasite motility.

## Results

### TbUNC119 was identified by comparative analysis

By comparing *T. brucei* whole gene products (amino acid sequence) to *C. elegans* UNC proteins, 88 genes were found whose products were highly similar to 11 *C. elegans* UNC proteins. We designated this group of gene products as TbCEUN (Table 1). Approximately two thirds of the TbCEUN gene products were kinesin-related molecules (Table 1). We also identified a trypanosome ortholog of UNC119 in the TbCEUN (Tb927.2.4580), and designated it as TbUNC119. TbUNC119 is a pathobiologically interesting molecule, because it has been reported that UNC119 has various functions and is conserved among various species including metazoans [13], [15], [16], [17]. Thus, in the present study, TbUNC119 was selected for further analysis in relation to parasite motility.

**Table 1:** TbCEUN (*T. brucei* gene products which have a high degree of similarity to *C. elegans* UNC proteins).

*C. elegans* UNC	Gene annotation	E-value*	*T. brucei* genes
UNC2	calcium channel α subunit	7.00E-50	Tb10.70.4750
UNC18	*Saccharomyces cervisiae* SEC1ortholog	1.00E-47	Tb09.160.0780
UNC22	Twitchin	1.00E-41	Tb927.7.6220
UNC43	type II calcium/calmodulin-dependent protein kinase (CaMKII) ortholog	1E-53∼5E-40	Tb10.70.1760, Tb927.8.870, Tb10.05.0210, Tb927.3.4560, Tb927.4.3770, Tb10.05.0200, Tb927.7.6220, Tb10.389.0490, Tb09.211.2360, Tb10.70.3410
UNC54	muscle myosin class II heavy chain (MHC B)	4E-92∼3E-40	Tb927.4.3380, Tb11.01.7990, Tb11.52.0008
UNC68	ryanodine receptor ortholog	3.00E-45	Tb927.8.2770
UNC78	actin-interacting protein 1 (AIP1) ortholog	2.00E-70	Tb927.3.4660
UNC82	ATP binding, protein kinase activity, serine/threonine kinase activity	5E-66∼1E-41	Tb10.70.1760, Tb927.3.4560, Tb927.8.870, Tb10.05.0210, Tb10.05.0200, Tb11.02.2050, Tb11.01.0330, Tb927.2.1820, Tb927.7.6220
UNC104	kinesin-like motor protein homologous	2E-90∼2E-40	Tb11.02.0090, Tb927.7.3830, Tb927.5.2090, Tb927.7.3000, Tb10.61.1020, Tb927.3.2040, Tb927.8.2630, Tb927.3.4960, Tb10.61.0990, Tb11.01.5490, Tb10.61.1750, Tb927.1.1350, Tb927.3.3390, Tb927.5.2410, Tb927.3.3400, Tb927.4.2730, Tb927.7.7260, Tb927.8.4950, Tb927.6.1770, Tb927.7.7120, Tb10.70.7260, Tb10.70.6990, Tb11.02.4260, Tb11.02.0400, Tb927.7.7120, Tb09.160.2260, Tb11.01.0850, Tb927.7.4830, Tb11.02.0790, Tb927.4.3910, Tb927.8.8350
UNC116	kinesin-1 heavy chain ortholog	6E-78∼6E-40	Tb927.1.1350, Tb927.5.2090, Tb10.61.1750, Tb11.02.0090, Tb11.01.5490, Tb927.3.2040, Tb927.3.3390, Tb927.4.2730, Tb927.7.3830, Tb927.3.3400, Tb927.7.7260, Tb927.6.1770, Tb927.3.2020, Tb10.70.7260, Tb927.6.4390, Tb927.5.2410, Tb11.02.4260, Tb10.61.1020, Tb11.02.0400, Tb10.61.0990, Tb927.7.3000, Tb927.3.4960, Tb927.8.2630, Tb927.7.5650, Tb11.01.0850, Tb927.4.3910, Tb927.8.8350, Tb11.02.2970, Tb11.01.3990
UNC119	adaptor protein	5.00E-46	Tb927.2.4580

*T. brucei hits with an expected value of 1×10^−40^ or less were retained.

A full-length TbUNC119 gene (cDNA) was cloned by PCR. The cDNA was 591 nt in length and had an ORF of 196 amino acids. The calculated molecular mass of the gene product was 22.7 kDa. A sequence homology search with the BLASTP program revealed that the sequence of TbUNC119 was from 42 to 61% identical to UNC119 sequences from a number of organisms, including *Trypanosoma cruzi* (61% identity), *Leishmania infantum* (57%), *Chlamydomonas reinhardtii* (45%), *C. elegans* (43%), and *Homo sapiens* (42%). The deduced amino acid sequence of TbUNC119 contained the conserved GMP PDE delta (cGMP phosphodiesterase delta subunit) domain found in other UNC119 proteins. TMpred analysis predicted that TbUNC119 had one transmembrane region spanning amino acid residues 12 to 31.

### Knock-down analysis of TbUNC119 showed no significant phenotype

**Figure 1:**
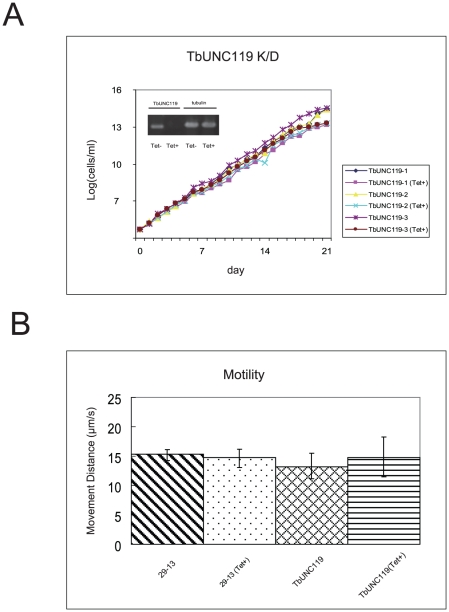
Knock-down analysis of TbUNC119. A. Growth of TbUNC119 knock-down procyclic form cells. Three TbUNC119 knock-down inducible cell lines were cloned (TbUNC119-1, TbUNC119-2 and TbUNC119-3). RNAi-mediated gene knock-down was induced with tetracycline. (Tet+) denotes RNAi-induced cells. There was no significant difference in the growth of the cell lines with or without RNAi induction. The inset shows TbUNC119 RT-PCR results with tubulin as a control. B. Motility assay of TbUNC119 knock-down cells. TbUNC119 knock-down cells and parental 29-13 cells were assayed for motility 7 days after RNAi induction or without RNAi induction. Thirty seconds of motion was captured for each cell. Motility traces for 50 cells in each group were generated using Videopoint 2.5 software. Cells that were not tracked for the full 30 s were not used for the analysis. There was no significant difference in the motility of the cell lines with or without RNAi induction.

To analyze the physiological function of TbUNC119, we first attempted to delete TbUNC119 genes with hygromycin-resistant and neomycin-resistant markers. However no transformants were obtained. Thus we then performed RNAi-mediated gene knock-down analysis. For these studies, we used *T. brucei* procyclic 29-13 cells, in which RNAi can be induced by the addition of tetracycline. RT-PCR analysis 7 days after RNAi induction revealed marked reduction of TbUNC119 transcripts (Fig. 1A). Western blot analysis also showed marked reduction of TbUNC119 in TbUNC119 knock-down cells 7 days after RNAi induction (Fig. S1). However, there was no significant change in growth between RNAi-induced TbUNC119 knock-down cells and RNAi-uninduced control cells (Fig. 1A). A motility assay also showed no significant change between the RNAi-induced TbUNC119 knock-down cells and RNAi-uninduced control cells (Fig. 1B).

### Identification of the TbUNC119 binding protein

**Figure 2:**
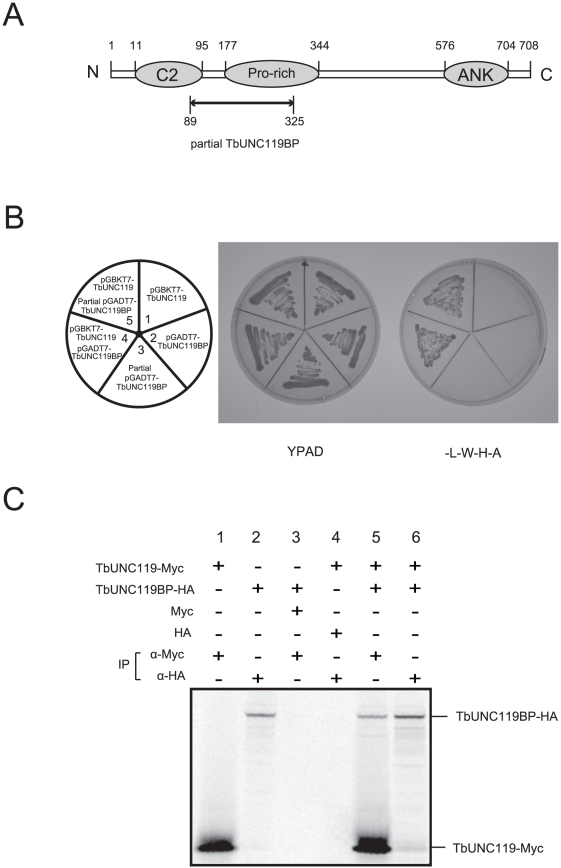
TbUNC119 interacted with TbUNC119BP (Tb927.7.5300). A. Schematic representation of the molecular structure of TbUNC119BP. Numbers represent amino acid residues. C2, Pro (proline)-rich, and ANK (ankyrin) repeat motifs were found between residues 11 and 95, between residues 177 and 344, and between residues 576 and 704, respectively, in the TbUNC119BP molecule. Arrow indicates a region which was harbored in the prey plasmid (Fig. 2B). B. Yeast two-hybrid analysis of TbUNC119 interaction. Yeast strain AH109 was co-transformed with pGBKT7-TbUNC119 and pGADT7-TbUNC119BP (full-length or partial; Fig. 2A). Growth of transformants on media -L-W-H-A (without leucine, tryptophan, histidine, and adenine) indicates protein-protein interaction (sectors 4 and 5). Yeast cells transformed with each plasmid failed to grow on media -L-W-H-A (sectors 1, 2 and 3). C. Co-immunoprecipitation of TbUNC119 and TbUNC119BP. ^35^S-labeled Myc-tagged TbUNC119 and ^35^S-labeled HA-tagged TbUNC119BP were expressed *in vitro* using the TNT T7 Quick coupled transcription/translation system and ^35^S-methionine. Lysates were precipitated on anti-Myc agarose or anti-HA agarose. Immunoprecipitated samples were separated by electrophoresis.

Since RNAi-mediated knock-down of TbUNC119 did not result in any characteristic phenotypes, we searched for molecules that may interact or associate with TbUNC119 using yeast two-hybrid analysis. In this analysis, TbUNC119 cDNA was fused to a bait vector, and a *T. brucei* cDNA library was constructed and fused to a prey vector. After transformation of AH109 yeast cells using these vectors, four colonies were obtained on four drop-out plates (SD/-ade/-his/-leu/-trp). Sequence analysis of the recovered prey plasmids revealed that while the genes encoding the plasmids in two of the colonies were not in frame, the other two colonies harbored the same prey plasmid that partially encoded Tb927.7.5300 (89 to 325 aa) in frame, including most of the proline-rich region of the molecule (Fig. 2A). The plasmid partially encoding Tb927.7.5300 was co-transformed into the host strain together with the TbUNC119 bait plasmid. The transformed yeast cells grew on the four drop-out plates, confirming *in vivo* interaction between TbUNC119 and Tb927.7.5300 (Fig. 2B). Tb927.7.5300 was thus designated as TbUNC119 binding protein (TbUNC119BP).

A full-length TbUNC119BP gene (cDNA) was cloned by PCR. The cDNA was 2127 nt in length with an ORF of 708 amino acids. The calculated molecular mass of the gene product was 73.1 kDa. A TbUNC119BP full-length encoding prey plasmid was co-transformed into the host strain together with the TbUNC119 bait plasmid. The transformed yeast cells grew on the four drop-out plates, further confirming *in vivo* interaction between TbUNC119 and TbUNC119BP (Fig. 2B).

A BLASTP sequence homology search revealed that the TbUNC119BP gene had a high degree of similarity only to kinetoplastida genes: *T. brucei gambiense* (98%), *T. cruzi* (48%), and *L. infantum* (52%). The deduced amino acid sequence of TbUNC119BP contained C2, Pro (proline)-rich and ANK (ankyrin) repeat domains (Fig. 2A). TMpred analysis predicted that TbUNC119BP had one transmembrane region spanning amino acid residues 34 to 53.

To further confirm the association of TbUNC119 and TbUNC119BP, an immunoprecipitation assay was performed using the TNT coupled Reticulocyte Lysate System. As shown in Fig. 2C, TbUNC119-Myc bound efficiently to TbUNC119BP-HA (lane 5), but not to the HA-tag alone (lane 3). In contrast, TbUNC119BP-HA bound efficiently to TbUNC119-Myc (lane 6), but not to the Myc-tag alone (lane 4). In addition, although the excess amount of TbUNC119-Myc existed in the lysate, TbUNC119BP-HA bound to almost equal (molar) amount of TbUNC119-Myc (Fig. 2C, lane 6), suggesting specific interaction between TbUNC119 and TbUNC119BP: the relative intensities of the two bands in lane 6 correspond to the ratio of the molecular weights. Our results hence demonstrated that TbUNC119 interacted with TbUNC119BP *in vitro*.

**Figure 3:**
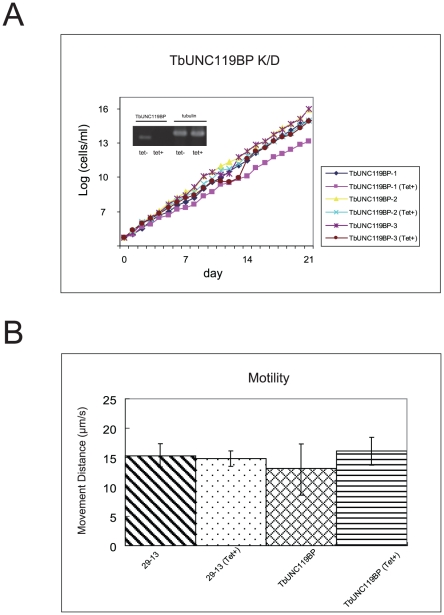
Knock-down analysis of TbUNC119BP. A. Growth of TbUNC119BP knock-down procyclic form cells. Three TbUNC119BP knock-down inducible cell lines were cloned (TbUNC119BP-1, TbUNC119BP-2 and TbUNC119BP-3). RNAi-mediated gene knock-down was induced with tetracycline. (Tet+) denotes RNAi-induced cells. There was no significant difference in the growth of the cell lines with or without RNAi induction. The inset in A shows TbUNC119BP RT-PCR results with tubulin as a control. B. Motility assay of TbUNC119BP knock-down cells. TbUNC119BP knock-down cells and parental 29-13 cells were assayed for motility 7 days after RNAi induction or without RNAi induction. Thirty seconds of motion was captured for each cell. Motility traces for 50 cells in each group were generated using Videopoint 2.5 software. Cells that were not tracked for the full 30 s were not used for the analysis. There was no significant difference in the motility of the cell lines with or without RNAi induction.

We then performed RNAi-mediated knock-down of the TbUNC119BP gene. RT-PCR analysis conducted 7 days after RNAi induction demonstrated marked reduction of TbUNC119BP transcripts (Fig. 3A), while western blot analysis showed no significant change of TbUNC119 in TbUNC119BP knock-down cells 7 days after RNAi induction (Fig. S1). However, there was no significant change in growth between RNAi-induced TbUNC119BP knock-down cells and RNAi-uninduced control cells (Fig. 3A). A motility assay also showed no significant change between RNAi-induced TbUNC119BP knock-down cells and RNAi-uninduced control cells (Fig. 3B).

### TbUNC119-TbUNC119BP double knock-down led to apoptosis, reduced motility, and abnormal cellular morphology in *T. brucei*

**Figure 4:**
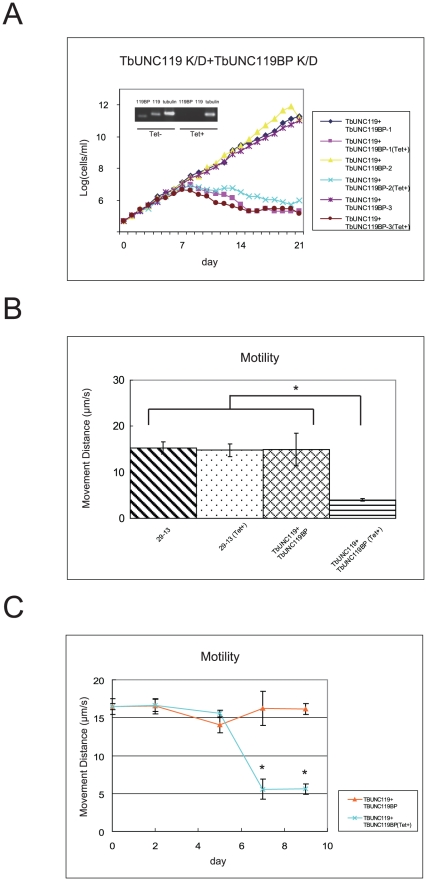
Double knock-down analysis of TbUNC119 and TbUNC119BP. A. Growth of TbUNC119-TbUNC119BP double knock-down procyclic form cells. Three TbUNC119 and TbUNC119BP double knock-down inducible cell lines were cloned (TbUNC119+TbUNC119BP-1, TbUNC119+TbUNC119BP-2 and TbUNC119+TbUNC119BP-3). RNAi-mediated gene knock-down was induced with tetracycline. (Tet+) denotes RNAi-induced cells. The growth rates of the double knock-down cells were significantly reduced, and the cells could not grow after 12 days of RNAi induction. The inset in A shows TbUNC119 and TbUNC119BP RT-PCR results with tubulin as a control. B. Motility assay of TbUNC119-TbUNC119BP double knock-down cells. Double knock-down cells and parental 29-13 cells were assayed for motility 7 days after RNAi induction or without RNAi induction. Thirty seconds of motion was captured for each cell. Motility traces for 50 cells in each group were generated using VIDEOPOINT 2.5 software. Cells that were not tracked for the full 30 s were not used for the analysis. Motility of the RNAi-induced double knock-down cells was significantly reduced (*: *P*<0.01). C. Time-course motility assay of TbUNC119-TbUNC119BP double knock-down cells. Double knock-down cells were assayed for motility 0, 2, 5, 7 and 9 days after RNAi induction or without RNAi induction. Thirty seconds of motion was captured for each cell. Motility traces for 50 cells in each group were generated using VIDEOPOINT 2.5 software. Cells that were not tracked for the full 30 s were not used for the analysis. Motilities of the double knock-down cells 7 days and 9 days after RNAi induction were significantly reduced (*: *P*<0.01).

We next investigated whether double knock-down of TbUNC119 and TbUNC119BP lead to changes in *T. brucei* phenotypes. Genes for TbUNC119 and TbUNC119BP were cloned into the knock-down vector with which *T. brucei* cells were transformed. RT-PCR analysis 7 days after RNAi induction resulted in marked reduction of both TbUNC119 and TbUNC119BP transcripts (Fig. 4A). Western blot analysis also showed marked reduction of TbUNC119 in double knock-down cells 7 days after RNAi induction (Fig. S1). The growth rate of RNAi-induced double knock-down cells was significantly reduced, and the cells were unable to grow after 12 days of RNAi induction (Fig. 4A).

Motility of RNAi-induced double knock-down cells was compared to that of RNAi-uninduced control cells. As shown in Fig. 4B, the traced movement distance of RNAi-induced double knock-down cells was 4.02±1.12 µm/s, while that of RNAi-uninduced control cells was 14.94±8.66 µm/s. The motility was thus significantly reduced in RNAi-induced double knock-down cells (*P*<0.01). Then we conducted time-course motility assay to investigate when the motility reduction occurred in RNAi-induced double knock-down cells. Motilities of double knock-down cells 0, 2, 5, 7 and 9 days after RNAi induction were compared to those of RNAi-uninduced control cells. The reduced motility of double knock-down cells was observed from 7 days after RNAi induction (Fig. 4C).

**Figure 5:**
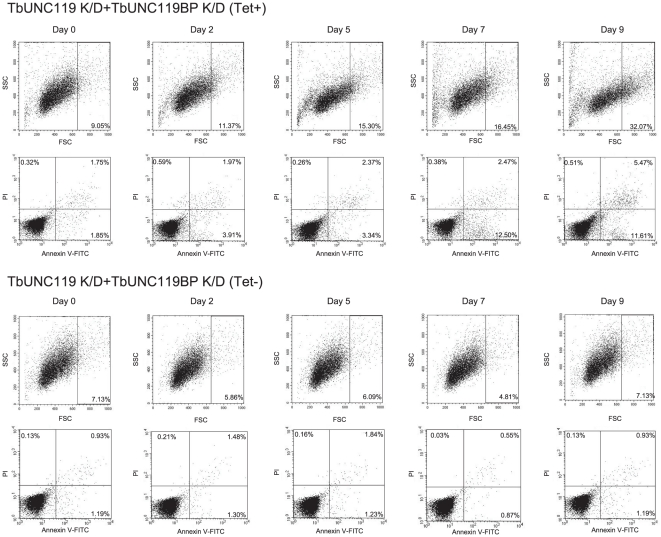
Flow cytometry analysis of TbUNC119-TbUNC119BP double knock-down cells. 2×10^5^ TbUNC119-TbUNC119BP double knock-down cells 0, 2, 5, 7 and 9 days after RNAi induction, and 2×10^5^ RNAi-uninduced control cells were harvested, washed with PBS, and stained with both Annexin V-FITC and propidium iodide (PI). (Tet+) denotes RNAi-induced cells, while (Tet-) denotes RNAi-uninduced control cells. Size-increased cells are shown as cell populations (%) in each Forward Scatter (FSC) measurement. Apoptotic cells are shown as cell populations (%) with Annexin V-FITC-positive and PI-negative. The proportions of size-increased cells in RNAi-induced group constituted 32.07% at day 9, while those in the RNAi-uninduced control cells were less than 7.13% at each time point. The proportions of apoptotic cells in RNAi-induced group constituted 12.50% at day 7 and 11.61% at day 9, while those in the RNAi-uninduced control cells were less than 1.30% at each time point.

To further analyze the mechanisms underlying reduced cell growth, annexin V-stained flow cytometry analysis was performed. Double knock-down cells were harvested 0, 2, 5, 7 and 9 days after RNAi induction along with RNAi-uninduced control cells. Flow cytometry analysis revealed increase in apoptotic cell ratios in double knock-down cells from 2 days after RNAi induction and apoptotic cells constituted 12.50% of the double knock-down cells 7 days after RNAi induction, while less than 1.30% of the RNAi-uninduced control cells were apoptotic (Fig. 5). In addition, Forward Scatter (FSC) distributions suggested that the RNAi-induced double knock-down cells had increased in size and those cells constituted 32.07% in double knock-down cells 9 days after RNAi induction, while less than 7.13% of the RNAi-uninduced control cells had increased in size (Fig. 5).

**Figure 6:**
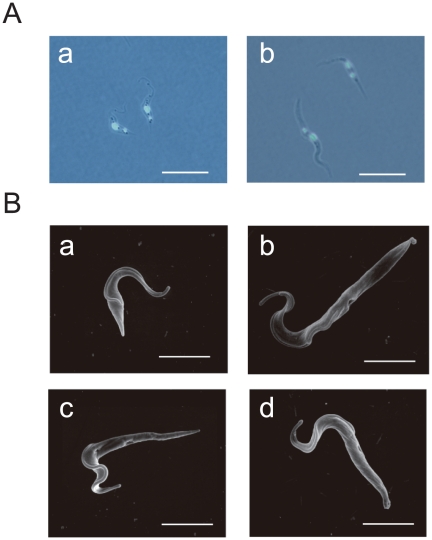
The morphology of TbUNC119-TbUNC119BP double knock-down cells. A. Fluorescent differential interference contrast image of TbUNC119-TbUNC119BP double knock-down procyclic form cells. TbUNC119-TbUNC119BP double knock-down cells 7 days after induction (b) and RNAi-uninduced control cells (a) were labeled with DAPI. Note that the posterior end and the kinetoplast were extended in the RNAi-induced double knock-down cells. The phenotype was clearly observed from 5 days after RNAi induction in double knock-down cells. Bar = 20 µm. B. Scanning electron microscopy of TbUNC119-TbUNC119BP double knock-down procyclic form cells. Double knock-down cells 7 days after RNAi induction (b–d) and RNAi-uninduced control cell (a) were used for the analysis. Bar = 10 µm.

While counting the numbers of double knock-down cells, we observed abnormally extended trypanosomes in each time point clearly from 5 days after RNAi induction, which were consistent with flow cytometry results (Fig. 5). Fluorescent microscopy and scanning electron microscopy analyses revealed that the phenotype was caused by extension mainly in the region between the posterior end and the kinetoplast, while knock-down had no significant effect on the length between the kinetoplast and the nucleus, or between the nucleus and the anterior end (Fig. 6).

### TbUNC119 was localized on the flagellum

**Figure 7:**
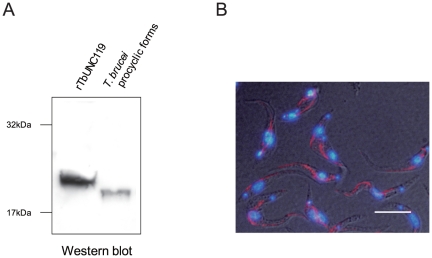
Expression profile of TbUNC119. A. Western blot analysis of TbUNC119. rTbUNC119 (0.5 µg) and whole cell extract of *T. brucei* procyclic forms (5 µg) were separated by electrophoresis, transferred onto a PVDF membrane, and probed with 2000-fold diluted anti-rTbUNC119 mouse polyclonal antibody. The membrane was subsequently probed with 2000-fold diluted peroxidase-labeled purified antibody to mouse IgG. B. Differential interference contrast image of procyclic forms cells. The 29-13 procyclic form cells were stained with DAPI and 1000-fold diluted anti-rTbUNC119 mouse polyclonal antibody, followed by 2000-fold diluted XRITC-labeled anti-mouse IgG. Bar = 20 µm.

To determine the cellular localization of TbUNC119, rTbUNC119 protein was expressed, purified, and used to immunize mice. The resulting polyclonal anti-rTbUNC119 antibody (sera) recognized TbUNC119 molecule, producing a single band in western blot analysis of *T. brucei* procyclic form cell extracts (Fig. 7A). The difference of molecular size between rTbUNC119 and TbUNC119 was considered to be due to N-terminal additional peptides, including 6 X His-tag in rTbUNC119, which were encoded in the expression vector (11 aa; the calculated molecular mass was 1.5 kDa). Procyclic form cells were then stained with this antibody and DAPI. As shown in Fig. 7B, TbUNC119 was localized on the flagellum.

The localization of TbUNC119 was also investigated by biochemical fractionation. *T. brucei* procyclic form cell extracts were fractionated into cytosol and flagellum fractions. Subsequent western blot analysis using the polyclonal anti-rTbUNC119 antibody demonstrated predominant expression of TbUNC119 in flagellum fraction, confirming its flagellum localization (Fig. S2).

## Discussion

Motility plays an important role in pathogen biology, host-pathogen interactions, and disease pathogenesis. The contribution of pathogen motility to the virulence of bacterial pathogens has been well documented [18], [19]. Recent studies also demonstrated a role for motility in the ability of protozoan parasites to infect mammalian hosts and/or vectors [20], [21], [22], [23].

*T. brucei* is a highly motile parasite, and coordinated motility is crucial for its development in the tsetse fly vector as well as disease pathogenesis in mammalian hosts [3], [24], [25]. Migration between the gut and salivary glands of the tsetse fly is essential for stage development [3], [4]. If motility is compromised in bloodstream trypanosomes, cytokinesis fails, and a lethal phenotype results [2]. We used procyclic (insect) form cells to study parasite motility during stage development in the hope of finding novel motility-related molecules.

Since *C. elegans* lacks a flagellum, its genome was used in a previous *in silico* study to subtract genes unrelated to the flagellum from the *T. brucei* genome [6]. In our study, the 88 *T. brucei* gene products (amino acid sequence) highly similar to *C. elegans* UNC proteins were identified (TbCEUN). Two thirds of these gene products were kinesin-related molecules, which were believed to associate with microtubules, the structural components of flagella. Thus, although *C. elegans* lack flagellum, presumably some of the TbCEUN molecules, such as TbUNC119, could be flagellum proteins with evolutionarily distinct functions from analogous proteins in *C. elegans*.

A number of metazoan species and other organisms, including kinetoplastida, *Naegleria* and *Chlamydomonas*, share the UNC119 molecule [13], [15], [16], [17]. HsUNC119 is a signaling molecule that associates with interleukin (IL)-5 receptor α and Src family tyrosine kinases, and is essential for T cell activation [13], [26]. Since IL-5 receptor α is present only in the vertebrate immune system, a unique HsUNC119-mediated signal transduction system can be conferred by the associated IL-5 receptor α, and not by HsUNC119 alone. Similarly, since UNC119 is conserved among various species, UNC119-mediated, species-specific function(s) may be conferred by its associated protein. This hypothesis is plausible because TbUNC119BP is a kinetoplastida-specific molecule. Moreover, in terms of molecular structure, similarity to kinetoplastida molecules other than African trypanosome species was restricted to merely part of this molecule. TbUNC119BP is therefore considered to be an African trypanosome-specific molecule.

TbUNC119 was considered to interact with TbUNC119BP at the proline-rich domain of TbUNC119BP (Fig. 2A). Although experimental verification is needed, bioinformatic analyses predicted that TbUNC119BP was a membrane protein with a C2 domain that appears to be involved in targeting proteins to the cell membrane [27]. In addition, the ankyrin repeats found in TbUNC119BP are believed to mediate protein-protein interactions [28]. Thus, the TbUNC119-TbUNC119BP complex, together with other associating protein(s), may regulate cell motility, propagation, and morphology through an unknown signal transduction system. Since the flagellum has been recognized as a good model for understanding signal transduction in *T. brucei* [25], discovery of molecules that associate with the TbUNC119-TbUNC119BP complex would reveal the novel signal transduction mechanism characteristic to *T. brucei*.

In our study, TbUNC119-TbUNC119BP double knock-down analysis resulted in characteristic phenotypes, including cellular apoptosis, reduced motility, and morphological change. Among those phenotypes, cellular apoptosis and morphological change were observed prior to reduced motility in RNAi-induced double knock-down cells (Fig. 4B and Fig. 5). However, RNAi-mediated depletion of either of those molecules resulted in no apparent phenotype. One possible explanation for this lack of phenotype is that when expression-level of one molecule was decreased, signals that passed through the other molecule might have been complementary enhanced. Alternatively, knock-down of each molecule may not have completely eliminated the target gene expression while the RT-PCR analyses demonstrated the marked reduction of both transcripts and western blot analyses demonstrated the marked reduction of TbUNC119 molecule (Fig. 1A, Fig. 3A and Fig. S1).

Both cellular length and motility are controlled in a stage-dependent manner in the tsetse fly vector [4], [25]. In the midgut of tsetse fly, the trypanosome population is tightly maintained by apoptosis in order for the organism to adapt to limited sources of proline as an energy source [29]. The TbUNC119-TbUNC119BP molecule may be involved in this control system, and failure of the control may have cleared stage-dependent gene expression. Thus several phenotypes that were infrequently observed in the procyclic stage, such as reduced motility, extended cell shape, and apoptosis, may have come out. In particular, the extended region of the RNAi-induced double knock-down cells was primarily between the posterior end and the kinetoplast (Fig. 6A, B). Several molecules that can cause similar stage-specific morphological phenotypes in trypanosomes have been identified. Examples include TbZFP2, which was identified as a member of a novel family of small proteins possessing a zinc finger motif, and predicted to bind RNA [30]. When TbZFP2 was ectopically overexpressed in procyclic form cells, ‘nozzle formation’ was observed due to expansion of the kinetoplast-posterior dimension of the cell [30]. Since TbUNC119 was localized along the flagellum (Fig. 7 and Fig. S2), TbUNC119-TbUNC119BP molecules may play an important role in flagellum-mediated stage progression. Alternatively, since UNC119 molecules in other organisms inhibit endocytosis [31], [32], RNAi-induced double knock-down cells may not have been able to control endocytosis; accelerated endocytosis therefore may have led to abnormally extended cell shape, reduced motility, and eventual apoptosis.

Further analyses of both TbUNC119 and TbUNC119BP molecules not only in procyclic but also in bloodstream trypanosomes together with analyses of other motility-related molecules found in this study, would provide a clearer picture of the relationship between parasite motility and stage progression. In addition, motility-related molecules might prove to be promising targets for developing novel drugs to treat African sleeping sickness.

## Materials and Methods

### Bioinformatic analysis

Genome sequence data and predicted gene information were imported from public databases. Information for *T. brucei* genes was collected from GeneDB.org as the *T. brucei* whole protein database. Information regarding *C. elegans* UNC proteins was collected from WormBase as the *C. elegans* UNC database. Databases were stored in different tables and managed by MySQL. The similarity search script was written in Perl, taking advantage of the stand-alone blast2 algorithm [33]. *T. brucei* hits with an expected value of 1×10^−40^ or less were retained. Transmembrane-region prediction was performed using web-based TMpred analysis (http://www.ch.embnet.org/software/TMPRED_form.html).

### Parasites and culture

*T. brucei* 29-13 (procyclic form) cells (generous gift from Dr. George A. M. Cross, Laboratory of Molecular Parasitology, Rockefeller University) were cultured in SDM-79 medium [34], supplemented with 10% (v/v) heat-inactivated fetal bovine serum and 7.5 µg/ml bovine hemin solution.

### Gene cloning

*T. brucei* total RNA was extracted using TRIzol reagent (Invitrogen), and subsequently reverse-transcribed with You-Prime first strand beads (GE). The resulting *T. brucei* cDNA was used as a template in PCR experiments. Primers, 5′-ATGGCTGCGACGACCGAGGTCAC-3′ and 5′-TTATTTAGTGAATTCCTCCTGGTG-3′, were designed to amplify the full-length TbUNC119 cDNA, while primers, 5′-ATGAATGTCGATGGTACTGTCC-3′ and 5′-TTACATTGTTTCAGCCAACATC-3′, were designed to amplify the full-length TbUNC119BP cDNA. PCR was carried out using the above primer sets with an initial 1 min denaturation at 94°C, followed by 30 cycles at 94°C for 15 sec, at 55°C for 30 sec, and at 72°C for 1 min. This was followed by a final extension of 5 min at 72°C. The resulting TbUNC119 and TbUNC119BP cDNAs were cloned into a TOPO vector (Invitrogen) and used for further analysis after confirmation of the sequence.

### Two-hybrid analysis

The TbUNC119 open reading frame (ORF) encoding region was sub-cloned into pGBKT7 bait vector (pGBKT7-TbUNC119). A *T. brucei* two-hybrid library was constructed and fused to a pGADT7 prey vector using a Matchcmaker Library Construction and Screening Kit (Clontech) according to the manufacturer's protocol. Both the pGBKT7-TbUNC119 and pGADT7-*T. brucei* two-hybrid libraries were co-transformed into AH109 yeast cells. Transformants were selected on four drop-out SD/-Trp/-Leu/-His/-Ade culture plates after 7 days of incubation at 30°C, and the resulting positive clones were confirmed by X-gal overlay assay. The plasmids in the transformants were recovered as described previously [35], and then subjected to sequence analysis.

For confirmation of the screening results, the TbUNC119BP full-length amino acid encoding cDNA was sub-cloned into the *Eco*RI and *Bam*HI sites of the pGADT7 vector (pGADT7-TbUNC119BP). The pGADT7-partial TbUNC119BP (one of the recovered plasmids), pGADT7-TbUNC119BP, or pGADT7 were transformed into AH109 yeast cells together with pGBKT7-TbUNC119 or pGBKT7, and plated on SD/-ade/-his/-leu/-trp media.

### Immunoprecipitation

^35^S-labeled Myc-tagged TbUNC119 and ^35^S-labeled HA-tagged TbUNC119BP were expressed *in vitro* from pGBKT7-TbUNC119 and pGADT7-TbUNC119BP, respectively, using the TNT T7 Quick coupled transcription/translation system (Promega) and ^35^S-methionine (ICN) according to the manufacturer's protocol. ^35^S-labeled Myc-tagged TbUNC119 and ^35^S-labeled HA-tagged TbUNC119BP were immunoprecipitated using the Matchmaker Co-IP kit (Clontech) according to the manufacturer's protocol. Relative intensities for ^35^S-labeled Myc-tagged TbUNC119 and ^35^S-labeled HA-tagged TbUNC119BP were measured using ImageJ application according to the application manual [36]. The intensities were divided by each molecular weight to obtain relative molar amount.

### RNAi analysis

RNAi-mediated knock-down analyses of TbUNC119 and TbUNC119BP were performed as described previously [37]. TbUNC119 cDNA (nt 1 to 468) was amplified by PCR using primers, 5′-CTCGAGCCATTCTGCATTGAATTGCG-3′ and 5′-AAGCTTATGGCTGCGACGACCGAGGTC-3′, which contained restriction sites for *Xho*I and *Hind*III, respectively. TbUNC119BP cDNA (nt 9 to 528) was amplified by PCR using primers, 5′-CTCGAGCGATGGTACTGTCCAACAAGC-3′ and 5′-AAGCTTTTGGGAGAATGCCGTAAATG-3′, which also contained restriction sites for *Xho*I and *Hind*III, respectively. The PCR products were cloned into the *Xho*I/*Hind*III sites of the pZJM vector [38] (generous gift from Dr. Paul T. Englund, Johns Hopkins University School of Medicine).

For TbUNC119-TbUNC119BP double knock-down analyses, TbUNC119 cDNA (nt 1 to 468) was amplified by PCR using primers, 5′-CTCGAGCCATTCTGCATTGAATTGCG-3′ and 5′-CTCGAGATGGCTGCGACGACCGAGGTC-3′, which contained a restriction site for *Xho*I. TbUNC119BP cDNA (nt 9 to 528) was amplified by PCR using primers, 5′-CTCGAGCGATGGTACTGTCCAACAAGC-3′ and 5′-AAGCTTTTGGGAGAATGCCGTAAATG-3′, containing restriction sites for *Xho*I and *Hind*III, respectively. The amplified cDNA fragments were simultaneously cloned into the *Xho*I/*Hind*III sites of the pZJM vector. Transfection of plasmids into the 29-13 cells and induction of RNAi with tetracycline were performed as described previously [37]. Cell numbers were counted daily and plotted as growth curves. To check RNAi-mediated reduction of TbUNC119, TbUNC119BP and α-tubulin control expression, cells were collected at 7 days post-induction and subjected to RT-PCR analysis using the primer sets, (5′-CCATTCTGCATTGAATTGCG-3′ and 5′-ATGGCTGCGACGACCGAGGTC-3′), (5′-CGATGGTACTGTCCAACAAGC-3′ and 5′-TTGGGAGAATGCCGTAAATG-3′) and (5′-ATGCGTGAGGCTATCTGCATC-3′ and 5′-AGGTTGCGGCGAGTCAAATC-3′), respectively.

### Motility assay

TbUNC119 knock-down cells, TbUNC119BP knock-down cells, TbUNC119-TbUNC119BP double knock-down cells, and parental 29-13 cells were assayed for motility 7 days after RNAi induction or without RNAi induction. For time-course study, TbUNC119-TbUNC119BP double knock-down cells were assayed for motility 0, 2, 5, 7 and 9 days after RNAi induction or without RNAi induction. Digital images and movies were captured using an Olympus DP-20 digital camera system. Cells were observed using a 10× objective on an Olympus BX-40 microscope. Approximately 30 s videos for each slide were captured. Motility traces for 50 cells on each slide were generated using Videopoint 2.5 software. Cells that were not tracked for the full 30 s were not used for the analysis. Each motility assay was performed at each time point using three independently prepared samples. Student's *t*-Test was used to analyze statistical significance of the data.

### Flow cytometry analysis

TbUNC119-TbUNC119BP double knock-down cells (2×10^5^) were harvested 0, 2, 5, 7 and 9 days after RNAi-induction along with 2×10^5^ RNAi-uninduced control cells. Cells were washed with PBS and stained with both Annexin V-FITC and propidium iodide (PI), according to the manufacturer's protocol (Mebcyto Apoptosis Kit, MBL). Stained cells were analyzed using a flow cytometer (FACS calibur, Becton Dickinson). Each flow cytometry analysis was performed at each time point using at least two independently prepared samples to ensure the reproducibility of the results.

### Flagellum fractionation

Flagellum fractionation was performed as described previously [2]. Briefly, *T. brucei* procyclic form cells (5×10^6^) were treated with PEME (100 mM PIPES, 2 mM EGTA, 0.1 mM EDTA and 1 mM MgSO4; pH 6.9) +1% Nonidet P-40 substrate (WAKO) and subsequently treated with PEME +1 M NaCl in the presence of 10 µl protease inhibitor (SIGMA), 200 µg ml^−1^ DNase I (Nippon gene) and 50 µg ml^−1^ RNase A (Nippon gene) at 4°C for 10 min. Then flagellum (pellet) and cytosol (soluble) fractions were collected by centrifugation (16,000×g, 15 min, 4°C).

### Antibody

The TbUNC119 full-length amino acid encoding region was sub-cloned into the *Nde*I/*BamH*I sites of a pCold II expression vector (TAKARA). *E. coli* (C41) cells were transformed with pCold II/TbUNC119 and grown at 37°C. Expression was induced with 0.5 mM IPTG at 15°C when the OD_600_ reached 0.5. After overnight induction, cells were harvested and sonicated. The recombinant TbUNC119 (rTbUNC119) was subsequently purified using TALON metal affinity resin (Clontech), according to the manufacturer's protocol. Anti-rTbUNC119 antibody was raised in a mouse using the purified rTbUNC119 for first immunization and second boost.

### SDS PAGE and western blotting

Immunoprecipitated samples were separated on a 10% SDS-polyacrylamide gel. Whole cell extracts (5 µg), soluble (cytosol) fraction (5 µg) and pellet (flagellum) fraction (5 µg) of *T. brucei* procyclic form cells and rTbUNC119 (0.5 µg) were separated on 4-12% SDS-polyacrylamide gels and transferred onto PVDF membranes, which were then probed with 2000-fold diluted mouse polyclonal antibodies (sera) to rTbUNC119, as described previously [39]. The membranes were subsequently probed with 2000-fold diluted peroxidase-labeled purified antibody to mouse IgG (KPL), as described previously [39]. Signal detection was performed using TMB stabilized substrate for horseradish peroxidase (Promega) according to the manufacture's protocol.

### Scanning electron microscopy

At 7 days post-induction, TbUNC119-TbUNC119BP double knock-down cells were collected along with RNAi-uninduced control cells. Cells were analyzed by scanning electron microscopy as described previously [37].

### Fluorescent microscopy

*T. brucei* procyclic form cells were fixed and permeabilized for 1 h by incubating in methanol on poly-L-lysine-coated cover slips. The cells were then blocked for 1 h with 5% casein-containing PBS buffer, followed by 1 h incubation with mouse anti-rTbUNC119 antibody. After 3 washes with PBS, cells were incubated for 1 h with XRITC-conjugated anti-mouse IgG (Vector) and DAPI (SIGMA). Cover slips were then washed three times with PBS and mounted. Fluorescent images were obtained using an Olympus BX-40 microscope.

## Supporting Information

Figure S1 **Western blot analysis of TbUNC119 in knock-down cells.** TbUNC119 knock-down, TbUNC119BP knock-down, and TbUNC119-TbUNC119BP double knock-down *T. brucei* procyclic form cells were collected 0 and 7 days after RNAi induction (Tet+). Whole cell extracts (5 µg) of above cells were separated by electrophoresis, transferred onto PVDF membranes, and probed with 2000-fold diluted anti-rTbUNC119 mouse polyclonal antibody. The membranes were subsequently probed with 2000-fold diluted peroxidase-labeled purified antibody to mouse IgG.
(EPS)

Figure S2 **Flagellum-fractionation and western blot analysis of TbUNC119.** Soluble (cytosol) and pellet (flagellum) fractions were obtained from *T. brucei* procyclic form cells as described in materials and methods. Subsequently those fractions (5 µg) were separated by electrophoresis, transferred onto a PVDF membrane, and probed with 2000-fold diluted anti-rTbUNC119 mouse polyclonal antibody. The membrane was subsequently probed with 2000-fold diluted peroxidase-labeled purified antibody to mouse IgG. S denotes soluble (cytosol) fraction; P denotes pellet (flagellum) fraction.
(EPS)
